# Evaluation of the Antioxidant Activity of *Syzygium cumini* Leaves

**DOI:** 10.3390/molecules13102545

**Published:** 2008-10-16

**Authors:** Zhi Ping Ruan, Liang Liang Zhang, Yi Ming Lin

**Affiliations:** 1 Key Lab of Ministry of Education for Coast and Wetland Ecosystems, Xiamen 361005; P.R. China; E-mails: rzp20012001@yahoo.com.cn (Z-P. R.); zhll20086@gmail.com (L-L. Z.); 2 Department of Biology, School of Life Sciences, Xiamen University, Xiamen 361005; P.R. China

**Keywords:** *Syzygium cumini*, Antioxidant activity, Free radicals, High performance liquid chromatography

## Abstract

The antioxidant activity of *Syzygium cumini* leaf extracts was investigated using the 2,2-diphenyl-1-picrylhydrazyl (DPPH) free radical-scavenging and ferric-reducing antioxidant power (FRAP) assays. The methanolic extract and its four water, ethyl acetate, chloroform, and *n*-hexane fractions were prepared and subjected to antioxidant evaluation. The results showed that the ethyl acetate fraction had stronger antioxidant activity than the other ones. HPLC data indicated that *S*. *cumini* leaf extracts contained phenolic compounds, such as ferulic acid and catechin, responsible for their antioxidant activity. A significant linear relationship between antioxidant potency, free radical-scavenging ability and the content of phenolic compounds of leaf extracts supported this observation.

## Introduction

Consumption of fruits and vegetables is shown to lower the risk for chronic diseases such as cancer, cardiovascular diseases and stroke [1]. The positive health effects may be due to high contents of certain phenolic compounds in plant-derived foods [2]. Recently, phytochemicals and their effects on human health have been intensively studied. In particular, a search for antioxidants, hypoglycemic agents, and anticancer agents in vegetables, fruits, teas, spices and medicinal herbs has attracted great attention. 

*S*. *cumini* (L.) Skeels has been attributed in the Indian folklore medicine system to possess several medicinal properties [3]. The bark of the plant is astringent, sweet, refrigerant, carminative, diuretic, digestive, antihelminthic, febrifuge, constipating, stomachic and antibacterial. The fruits and seeds are used to treat diabetes, pharyngitis, spleenopathy, urethrorrhea and ringworm infection. The leaves have been extensively used to treat diabetes, constipation [4], leucorrhoea, stomachalgia, fever, gastropathy, strangury and dermopathy [3], and to inhibit blood discharges in the faeces [4]. The plant possesses acetyl oleanolic acid, triterpenoids, ellagic acid, isoquercitin, quercetin, kaempferol and myricetin in different concentrations [5]. Most of these compounds have been reported to possess antioxidant and free radical scavenging activities [6]. The chemical composition and antioxidant activity of *S*. *cumini* fruits have been studied recently [7, 8], but there is scant information about the antioxidant activity of *S*. *cumini* leaves. In this study, the total phenolic content of *S*. *cumini* leaves was determined, and their antioxidant properties were also evaluated.

## Results and Discussion

### Preparation of the methanolic extract and its fractions

Due to the complicated constituents and pharmacological diversities of plants, *in vitro* bioassay-guided fractionation has been effectively applied to screen the biological activities that contribute important indications for investigating the characteristics of active components [9]. The methanolic *S*. *cumini* leaf extract (ME) was fractionated by solvent–solvent partitioning to obtain four water (WtF), ethyl acetate (EaF), chloroform (CfF), and *n*-hexane (HxF) fractions. The recoveries of WtF, EaF, CfF, and HxF were about 37.33%, 5.33%, 4.01%, and 24.67%, indicating that the *S*. *cumini* leaf constituents belong mainly to the two opposing extremes of polarity.

### Total phenolic and total flavonoid content

Phenols are very important plant constituents because of their radical scavenging ability due to their hydroxyl groups [10]. The phenolics content may contribute directly to the antioxidative action [11]. It has been suggested that polyphenolic compounds have inhibitory effects on mutagenesis and carcinogenesis in humans [6]. Consequently, the antioxidant activities of plant/herb extracts are often explained by their total phenolics and flavonoid contents with good correlation. The total phenolic content in the methanolic extract of *S*. *cumini* was 610.32 ± 9.03 mg/g while the flavonoid content was 451.50 ± 9.85 mg/g. These results demonstrate that flavonoids represent the main group of phenolic compounds in *S*. *cumini* leaves.

### DPPH free radical-scavenging assay

The DPPH free radical is a stable free radical, which has been widely accepted as a tool for estimating free radical-scavenging activities of antioxidants [12]. The percentages of remaining DPPH in the presence of the ME and its fractions at different concentrations are shown in Table 1. 

**Table 1:** Remaining DPPH and ferric reducing power after addition of the methanolic extract (ME) of *S*. *cumini* leaves and its fractions (n = 3).

Concentration (μg/mL)	ME	HxF	WtF	EaF	CfF
DPPH *^a^*					
15.63	92.78 ± 0.50D	91.99 ± 0.37D	91.03 ± 0.61D	90.26 ± 3.55D	88.42 ± 1.08D
31.25	86.92 ± 0.78C	90.10 ± 0.18C	86.02 ± 1.82C	81.73 ± 0.43C	83.57 ± 0.98C
62.5	74.25 ± 0.45B	86.78 ± 0.84B	75.72 ± 1.67B	69.15 ± 0.30B	73.52 ± 0.39B
125	49.27 ± 3.35A	79.09 ± 0.67A	56.85 ± 2.66A	45.95 ± 0.72A	56.93 ± 1.02A
FRAP *^b^*					
15.63	0.13 ± 0.01a	0.06 ± 0.01a	0.10 ± 0.00a	0.11 ± 0.01a	0.11 ± 0.01a
31.25	0.19 ± 0.01b	0.07 ± 0.00a	0.16 ± 0.01b	0.20 ± 0.00b	0.18 ± 0.00b
62.5	0.33 ± 0.02c	0.10 ± 0.00b	0.27 ± 0.01c	0.35 ± 0.01c	0.31 ± 0.01c
125	0.60 ± 0.01d	0.15 ± 0.00c	0.51 ± 0.00d	0.68 ± 0.01d	0.55 ± 0.01d

*^a^* Data are presented as the percentage of remaining DPPH; *^b^* Data are presented as the absorbance at 593 nm; Different letters on the same column show significant differences from each other at *P* < 0.05; Statistical analysis was done by Duncan’s multiple range tests.

The proportions of the remaining DPPH decreased slightly with the WtF and CfF. Great decreases in a concentration-dependent manner of the remaining DPPH in ME and EaF indicated that with the exception of HxF, *S*. *cumini* extracts possess potent free radical-scavenging activity. By comparing the IC_50_ value of the ME and those of its active fractions with that of the authentic antioxidants, BHA and Vit. C (Table 2), it was found that the antioxidant activity of EaF (IC_50_: 112.79 µg/mL) was lower than that of Vit. C (IC_50_: 71.30 µg/mL), but not significantly different from that of BHA (IC_50_: 114.69 µg/mL) and ME (IC_50_: 125.39 µg/mL). By comparing ME and its active fractions, the free radical-scavenging activities followed the order: EaF ≈ ME > CfF ≈ WtF > HxF. The free radical-scavenging activity of the CfF, WtF and HxF were lower than that of EaF, which resulted from increasing the active components/units through condensation during the solvent–solvent partitioning processes. The results also indicated that the active components existed mainly in the medium-polarity EaF fraction. *S*. *cumini* contains quercetin and myricetin [8]. Flavonoids are well-known antioxidant constituents in plants [12]. *Spartium junceum*, a Turkish folk medicine, contains flavonoid glycosides, which possess potent antioxidant activity according to activity-guided fractionation [13]. Accordingly, the antioxidant activity of leaves of *S*. *cumini* may be related to its flavonoid constituents. A previous study has demonstrated that the leaf extract of *S*. *cumini* can protect against the radiation-induced DNA damage [14].

### Ferric reducing antioxidant power (FRAP)

The FRAP assay is based on the ability of antioxidants to reduce Fe^3+^ to Fe^2+^ in the presence of TPTZ, forming an intense blue Fe^2+^-TPTZ complex with an absorption maximum at 593 nm. The absorbance decrease is proportional to the antioxidant content [15]. All fractions showed high ferric reducing power with increasing concentration, with the exception of HxF (Table 1). At 125 µg/mL, the reducing power of the EaF (A_593_ = 0.68) was superior to that of ME (A_593_ = 0.60), CfF (A_593_ = 0.55) and WtF (A_593_ = 0.51). The FRAP value, expressed in ascorbic acid equivalents, was used to determine the antioxidant ability of the different extracts in present study. The FRAP values for the *S*. *cumini* leaf extracts were high, ranging from 2.32 ± 0.02 to 3.12 ± 0.07 mmol AAE/g (Table 2). In brief, the reducing power of ME and its active fractions exhibited the descending order of: EaF, ME, CfF and WtF.

**Table 2:** Antioxidant activities of the methanolic extract (ME) of leaves of *S*. *cumini* and its fractions using the (DPPH) free radical-scavenging assay and the (FRAP) ferric-reducing antioxidant power assay (n = 3).

Samples	Antioxidant activity	
	IC_50/DPPH_ (µg/mL) *^a^*	FRAP (mmol AAE/g) *^b^*
ME	125.39 ± 7.55b	2.73 ± 0.06d
HxF	479.56 ± 9.31d	0.53 ± 0.05a
CfF	149.33 ± 4.43c	2.51 ± 0.05c
EaF	112.79 ± 2.51b	3.12 ± 0.07e
WtF	150.66 ± 6.95c	2.32 ± 0.02b
VitC	71.30 ± 1.66a	--
BHA	114.69 ± 1.95b	4.66 ± 0.10f

*^a^* The antioxidant activity was evaluated as the concentration of the test sample required to decrease the absorbance at 517 nm by 50% in comparison to the control; *^b^* FRAP values are expressed in mmol ascorbic acid equivalent/g sample in dry weight; Different letters on the same column show significant differences from each other at *P* < 0.05; Statistical analysis was done by Duncan’s multiple range tests.

In addition, in a comparison of the ferric reducing power of the ME and its active fractions with BHA, the reducing power of all extracts were lower than that of BHA (4.66 mmol AAE/g). This could be explained that antioxidants are reducing agents. Antioxidants are compounds capable of donating a single electron or hydrogen atom for reduction. However, not all reducing agents are antioxidants. The good correlation was observed between the DPPH and FRAP assay, with the regression equation was *y* = 0.0096*x* – 0.1274 (*R* = 0.9905). The FRAP assay was found to be more sensitive than the DPPH free radical-scavenging assay, which is in agreement with the observation of Zheng and Wang [16].

### Correlations between total phenolic content and antioxidative function

**Figure 1:**
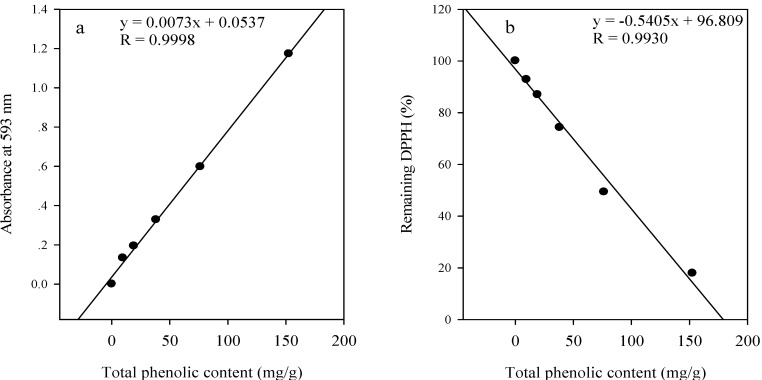
Relationships between the total phenolic content and the ferric reducing power (a); the total phenolic content and the free radical scavenging activity (b) of the methanolic extract (ME) of *S*. *cumini*.

The free radical scavenging activity and reducing power of the methanolic extract were significantly related to their total phenolic content (*R*_DPPH_ = 0.9930, *R*_FRAP_ = 0.9998) (Figure 1). The methanolic extract exhibited the highest radical scavenging activity and ferric reducing power with the greatest amount of phenolic content. The presence of polyphenolic compounds in methanolic extract of *S*. *cumini* might be responsible for this high antioxidant activity.

The extent of its antioxidant capacities were correlated with the contents of total phenolics and flavonoids. A linear regression analysis of the antioxidant activity with phenolic composition confirmed this observation. Benherlal and Arumughan [8] also observed that the seed ethanol extract of *S*. *cumini* had the extremely high antioxidant activity (IC_50__/DPPH_ of 8.6 ± 3.0 µg/mL).

**Figure 2:**
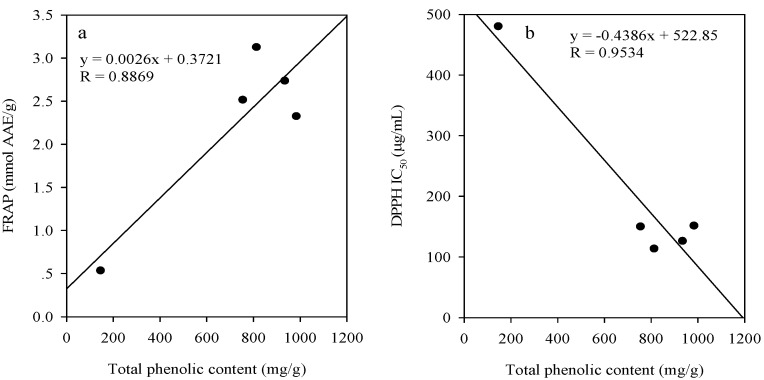
Relationships between the total phenolic content and the ferric reducing power (a); the total phenolic content and the free radical scavenging activity (b) of the five extracts of *S*. *cumini*.

In addition, the correlations between the total phenolic content of the five extracts and the free radical scavenging activity and reducing power were shown in Figure 2. The significant relationships between total phenolic contents of five extracts and the antioxidative activities suggest that phenolics might be the major antioxidant compounds in studied extracts.

### Determination of phenolic compounds

**Figure 3:**
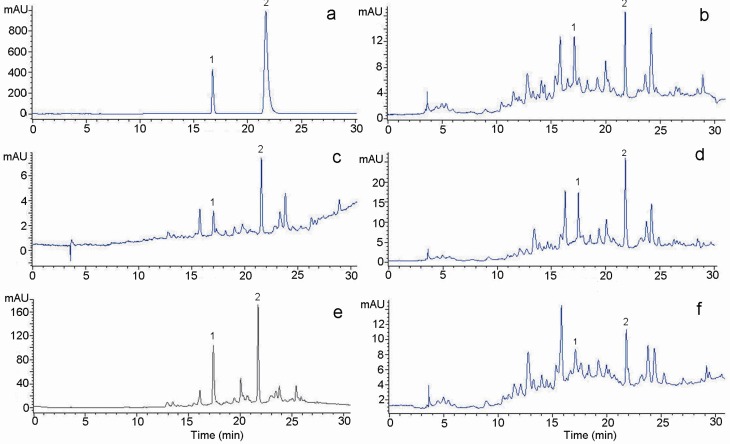
HPLC chromatogram (280 nm) of (a) authentic standards (1) catechin and (2) ferulic acid and (b) methanolic extract (c) *n*-hexane fraction (d) chloroform fraction (e) ethyl acetate fraction (f) water fraction of *S*. *cumini* leaves.

Phenolic compounds, such as quercetin, rutin, narigin, catechins, caffeic acid, gallic acid and chlorogenic acid, are very important plant constituents because of their antioxidant activities [17]. RP-HPLC coupled with UV–Vis DAD was employed to separate, identify and quantify phenolic compounds in the methanolic extract of *S*. *cumini* leaves and its fractions. The concentrations were determined by calculating the HPLC peak areas which are proportional to the amount of analytes in a peak and presented as the mean of three determinations which were highly repeatable. Figure 3a shows the chromatogram of authentic standards of catechin and ferulic acid. These phenolic compounds have been identified in the methanolic extract of *S*. *cumini* and its fractions according to their retention times and spectral characteristics of their peaks against those of standards (Figure 3b, c, d), as well as by spiking the samples with standards.

**Figure 4:**
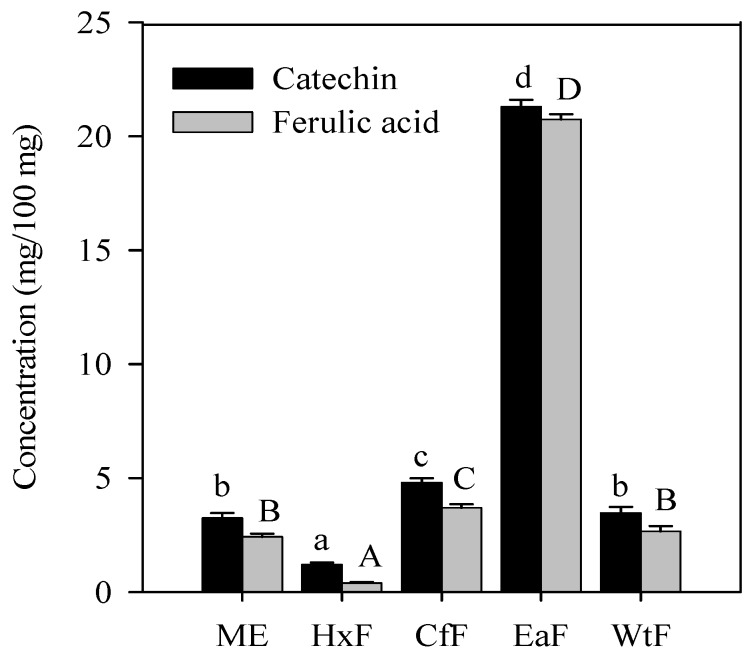
Concentrations of catechin and ferulic acid recovered from the methanolic extract (ME) of *S*. *cumini* leaves and its fractions. For catechin, different small letters show significant differences from each other at *P* < 0.05; for ferulic acid, different capital letters show significant differences from each other at *P* < 0.05.

The result from the chromatograms indicated that EaF contained the highest content of catechin and ferulic acid. As shown in Figure 4, EaF contained approximately 7-fold more catechin and about 9-fold more ferulic acid than ME. By comparing the different fractions, the content of catechin and ferulic acid decreased in the same order of EaF > CfF > WtF ≈ ME > HxF, and the rank order of ME and CfF was different according to their antioxidant potency and free radical-scavenging ability. This result indicates that, besides phenolic acids, the other complex phenolic compounds in *S*. *cumini* extracts may also be responsible for the antioxidant activity. The ethyl acetate solution is more suitable for the extraction of both catechin and ferulic acid. The higher yield of these compounds might contribute to the higher antioxidant activity of ethyl acetate fraction when compared with other fractions [18].

Catechin, a monomeric flavanol, is reported to have hydroxyl [19], peroxyl [20], superoxide [21] and DPPH [22] radical scavenging activities. Moreover, it can chelate iron [23]. Nakao *et al*. [24] found that ECG, epicatechin and catechin had a peroxyl radical scavenging activity ten times higher than L-ascorbate and β-carotene when tested on bacteria. Nanjo *et al*. [25] reported that DPPH radical scavenging activity of catechin and epicatechin was lower than EGC, ECG, and EGCG. Recently, catechins have been used as natural antioxidant in oils and fats against lipid oxidation, supplement for animal feeds both to improve animal health and to protect animal products, an antimicrobial agent in foodstuffs and a health functional ingredient in various foods and dietary supplements [26]. It was hypothesized that catechins might be localized near the membrane surface scavenging aqueous phase radicals [27] and preventing the consumption of α-tocopherol, whereas the latter mainly acts as a scavenger of lipid peroxyl radicals within the low-density lipoproteins. The study of the catechins is particularly important for the understanding of the antioxidant properties of teas, in which flavanols such as catechins and catechin/gallate esters constitute 26.7% of the solid content of freeze-dried green tea [28]. Ferulic acid and its precursors, *p*-coumaric acid and caffeic acid are synthesized in plants. Ferulic acid occurs in cereals and vegetables, such as rice, wheat, oats, tomatoes, asparagus, olives and many other plants. Recently, many studies have focused on the antioxidant potentialities of ferulic acid and its *n*-alkyl esters [29]. Sanchez-Moreno *et al*. [30] indicated that the inhibition of lipid oxidation of the phenolic compounds and antioxidant standards followed the order: rutin, ferulic acid > tannic acid, gallic acid, resveratrol > BHA, quercetin > tocopherol > caffeic acid, in a linoleic acid system. Meanwhile, the free radical-scavenging activity was in the order: gallic acid > tannic acid, caffeic acid, quercetin, BHA, rutin > ferulic acid, tocopherol, resveratrol. However, some studies indicated that ferulic acid was ineffective, and even promoted the oxidation of low density lipoprotein induced by copper [31]. According to our observation, catechin and ferulic acid may have important roles in the antioxidant activity and free radical scavenging ability of *S*. *cumini* leaf extracts.

## Conclusions

Total phenolic content in methanolic extract of *S*. *cumini* leaves was 610.32 ± 9.03 mg/g while the flavonoid content of the methanolic extract was 451.50 ± 9.85 mg/g. The five extracts of *S*. *cumini* leaves have potent antioxidant activity according to the DPPH and FRAP assays. The HPLC data indicated that *S*. *cumini* leaf extracts contained phenolic compounds, such as ferulic acid and catechin. The antioxidant activity of *S*. *cumini* leaf extracts may be related to their phenolic substrates. A significant linear relationship between antioxidant potency, free radical-scavenging ability and the content of phenolic compounds of leaf extracts supported this observation.

## Experimental

### Chemicals and plant materials

Vitamin C (Vit.C), butylated hydroxyanisole (BHA), tannic acid, ferulic acid, rutin, catechin, TPTZ (2,4,6-tripyridyl-S-triazine) and DPPH (2,2-diphenyl-1-picrylhydrazyl) were purchased from Sigma-Aldrich (St. Louis, MO). Milli-Q water and HPLC grade CH_3_CN for analytical RP-HPLC, and other chemicals were of analytical reagent (AR) purity grade. Mature leaves of *S*. *cumini* were collected at the campus of Xiamen University (Xiamen, P.R. China) and immediately freeze dried and ground.

### Extraction procedure

Leaf samples (120 g) were extracted by macerating them in methanol for 7 days at room temperature in a dark cabinet. After solvent evaporation in a rotary evaporator, the methanolic extract (ME) was further fractionated through solvent–solvent partitioning to obtain different fractions. The four solvents used for solvent–solvent partitioning to cover the range from high to low polarity were water, ethyl acetate, chloroform, and *n*-hexane. The ME and its four water (WtF), ethyl acetate (EaF), chloroform (CfF) and *n*-hexane (HxF) fractions were stored in an electronic dry cabinet protected from light with aluminum foil after solvent evaporation. 

### Determination of total phenolics and total flavonoid content

Total phenolics was measured with the Prussian blue method [32], using tannic acid as the standard. Total flavonoid content was determined according to a known method [33] with a slight modifications, the methanolic extract (2 mg) was added to 2 mL distilled water, followed by NaNO_2_ (0.3 mL, 5%). After 6 min at 25 °C, Al(NO_3_)_3_ (0.3 mL, 10%) was added. The reaction mixture was treated with NaOH (4 mL, 1 M) after 6 min, diluted to volume (10 mL) with 50% ethanol solution and, after a thorough mixing the absorbance at 500 nm was read. Rutin was used as the standard and the total flavonoid content was expressed as rutin equivalents (RE) mg/g dry weight of the extracts.

### Free radical scavenging ability on 2,2-diphenyl-1-picrylhydrazyl

To assess the scavenging ability on 2,2-diphenyl-1-picrylhydrazyl (DPPH), each extract (0.1 mL, 15–250 µg/mL) in methanol was mixed with methanol solution (3 mL) containing DPPH radicals (0.004%, w/w). The mixture was shaken vigorously and left to stand for 30 min in the dark before measuring the absorbance at 517 nm against a blank [34]. Then the scavenging ability was calculated using the following equation: Scavenging ability (%) = [(∆A_517_ of control – ∆A_517_ of sample)/∆A_517_ of control] × 100. Three replicates were carried out. 

### Ferric reducing/antioxidant power (FRAP) assay

The procedure was as described by Benzie and Strain [15]. Briefly, 3 mL of FRAP reagent, prepared freshly, was mixed with 0.1 mL of test sample, or methanol (for the reagent blank). The FRAP reagent contained 10 mM TPTZ solution (2.5 mL) in 40 mM HCl plus 20 mM FeCl_3_ (2.5 mL) and 0.3 M acetate buffer (pH 3.6, 25 mL). The absorbance of reaction mixture was measured spectrophoto-metrically at 593 nm after incubation at 25 °C for 10 min. FRAP assay records the change in absorbance at 593 nm owing to the formation of a blue colored Fe^II^-tripyridyltriazine compound from colorless oxidized Fe^III^ form by the action of electron donating antioxidants. All solutions were used on the day of preparation. The FRAP values, expressed in mmol ascorbic acid equivalents (AAE)/g sample in dry weight were derived from a standard curve. 

### HPLC analysis of phenolic compounds

The contents of phenolic compounds in leaf extracts of *S*. *cumini* were determined by HPLC, performed with an Agilent 1100 diode array detector system equipped with a quaternary pump. The analyses were carried out on a Hypersil ODS column (4.6 mm × 250 mm, 2.5 μm) column. Extracts were filtered through a 0.45 µm filter before use. Gradient B in A according to the elution profile 0-3 min 2% B (isocratic), 3-20 min 2%-25% B (linear gradient), 20-25 min 25%-35% B (linear gradient), (A) water (0.05% TFA), (B) CH_3_CN (0.05% TFA); flow rate 1 mL/min; volume injected 10 µL; temperature 22 °C; detection 280 nm. 

### Statistical analysis

All measurements were replicated three times and one-way analysis of variance (ANOVA) was used The Duncan’s multiple range tests were used to test the significant differences. All statistical analyses were performed with SPSS 11.0.
